# Particulars of Awards

**Published:** 1907-08-10

**Authors:** 


### PARTICULARS OF AWARDS.

### 33 GENERAL HOSPITALS.

Charing Cross Hospital, £1,496 1s. 8d.; French Hospital, £393 5s.; German Hospital, £660 16s. 8d.; Great Northern Central Hospital, £1,267 10s.; Guy's Hospital, £1,625; Hampstead General Hospital, £314 3s. 4d.; Italian Hospital, £216 13s. 4d.; Kensington General Hospital, £238 6s. 8d.; King's College Hospital, £1,457 1s. 8d.; London Hospital, £6,500; London Homœopathic Hospital, £482, 1s. 8d.; Phillips' Memorial Homœopathic Hospital, £49 16s. 8d.; London Temperance Hospital, £785 8s. 4d.; Metropolitan Hospital, £1,226 6s. 8d.; Mildmay Hospital, £325; Miller Hospital and Royal Kent Dispensary, £270 16s. 8d.; National Anti-Vivisection Hospital, nil; North-West London Hospital, £471 5s.; Poplar Hospital, £574 3s. 4d.; Royal Free Hospital, £1,370 8s. 4d.; St. George's Hospital, £2,275; SS. John and Elizabeth Hospital, £325; St. John's Hospital, Lewisham, £205 16s. 8d.; St. Mary's Hospital, £2,545 16s. 8d.; Seamen's Hospital Society, £1,635 16s. 8d.; The Middlesex Hospital and Convalescent Home, £2,757 1s. 8d.; Tottenham, Prince of Wales' General Hospital, £639 3s. 4d.; University College Hospital, £2,166 13s. 4d.; Walthamstow, etc., Hospital, £157 1s. 8d.; Wandsworth, Bolingbroke Hospital, £303 6s. 8d.; West Ham Hospital, £601 5s.; West London Hospital, £1,224 3s. 4d.; Westminster Hospital, £1,625.

### SPECIAL HOSPITALS.

5 Chest Hospitals.—City of London Hospital for Diseases of the Chest, Victoria Park, £812 10s.; Hospital for Consumption, Brompton, £3,011 13s. 4d.; Mount Vernon Consumption Hospital, Hampstead, £1,283 15s.; Royal Hospital for Diseases of the Chest, City Road, £568 15s.; Royal National Hospital for Consumption (Ventnor), £325.

18 Children's Hospitals.—Alexandra Hospital for Hip Disease, W.C., £444 3s. 4d.; Banstead Surgical Home, £28 3s. 4d.; Barnet Home Hospital, £52; Belgrave Hospital for Children, S.W., £227 10s.; Cheyne Hospital for Incurable Children, S.W., £151 13s. 4d.; East London Hospital for Children, Shadwell, E., £985 16s. 8d.; Evelina Hospital for Sick Children, Southwark, S.E., £270 16s. 8d.; Home for Incurable Children, Hampstead, £86 13s. 4d.; Home for Sick Children, Sydenham, S.E., £151 13s. 4d.; Hospital for Sick Children, Great Ormond Street, W.C., £1,083 6s. 8d.; Kensington, for Children, and Dispensary, £113 15s.; North-Eastern Hospital for Children, Hackney Road, N.E., £964 3s. 4d.; Paddington Green Hospital for Children, W., £352 1s. 8d.; St. Mary's Hospital, Plaistow, E., £308 15s.; St. Monica's Hospital, Brondesbury, N.W., £88 16s. 8d.; Victoria Hospital for Children, Chelsea, S.W., £747 10s.; Victoria Home, Margate, £46 6s. 8d.; Hospital for Hip Disease, Sevenoaks, £48 15s.

6 Lying-in Hospitals.—British Lying-in Hospital, Endell Street, W.C., £88 16s. 8d.; City of London Lying-in Hospital, City Road, E.C., £200; Clapham Maternity Hospital, £45 10s.; East End Mothers' Home, £86 13s. 4d.; General Lying-in Hospital, Lambeth, S.E., £56 6s. 8d.; Queen Charlotte's Lying-in Hospital, Marylebone Road, W., £557 18s. 4d.

6 Hospitals for Women.—Chelsea Hospital for Women, S.W., £341 5s.; Hospital for Women, Soho Square, W., £498 6s. 8d.; Grosvenor Hospital for Women and Children, Vincent Square, S.W., £195; New Hospital for Women, Euston Road, W.C., £346 13s. 4d.; Royal Waterloo Hospital for Children and Women, Lambeth, S.E., £195; Samaritan Free Hospital, Marylebone Road, W., £471 5s.

26 Other Special Hospitals.—Cancer Hospital, Brompton, S.W., nil; Middlesex Hospital, Cancer Wing, £292 10s.; London Fever Hospital, Islington, N., £325; Gordon Hospital for Fistula, Vauxhall Bridge Road, S.W., £43 6s. 8d.; St. Mark's Hospital for Fistula, City Road, E.C., £276 5s.; National Hospital for the Diseases of the Heart, Soho Square, W.,

£184 3s. 4d.; Female Lock Hospital, Harrow Road, W., £292 10s.; Hospital for Epilepsy, Paralysis, and other Diseases of the Nervous System, Maida Vale, W., £178 15s.; National Hospital for the Paralysed and Epileptic, W.C., £1,115 16s. 8d.; West End Hospital for Diseases of the Nervous System, £325; Central London Ophthalmic Hospital, Gray's Inn Road, W.C., £173 6s. 8d.; Royal Eye Hospital, St. George's Circus, S.E., £303 6s. 8d.; Royal London Ophthalmic Hospital, City Road, E.C., £1,191 13s. 4d.; Royal Westminster Ophthalmic Hospital, Charing Cross, W.C., £162 10s.; Western Ophthalmic Hospital, Marylebone Road, W., £108 6s. 8d.; Royal National Orthopædic Hospital, Great Portland Street, W., £119 3s. 4d.; Royal Sea Bathing Hospital, Margate, £200; St. John's Hospital for Diseases of the Skin, £54 3s. 4d.; Western Skin Hospital, Great Portland Street, W., nil; St. Peter's Hospital for Stone, Covent Garden, £113 15s.; Central London Throat and Ear Hospital, Gray's Inn Road, W.C., £46 11s. 8d.; Hospital for Diseases of the Throat, Golden Square, W., £16 5s.; London Throat Hospital, Great Portland Street, W., £27 1s. 8d.; Royal Ear Hospital, Frith Street, W., £43 6s. 8d.; Royal Dental Hospital of London, W.C., £400 16s. 8d.; National Dental Hospital, 149 Great Portland Street, W., £32 10s.

### 33 CONVALESCENT HOSPITALS.

Metropolitan Convalescent Institution, Walton, £438 15s.; Metropolitan Convalescent Institution, Broadstairs, £287 1s. 8d.; Metropolitan Convalescent Institution, Bexhill, £530 16s. 8d.; All Saints' Convalescent Hospital, Eastbourne, £433 6s. 8d.; All Saints' Convalescent Home, St. Leonards-on-Sea, £27 1s. 8d.; Ascot Priory Convalescent Home, £81 5s.; Brentwood Convalescent Home for Children, £10 16s. 8d.; Charing Cross Hospital Convalescent Home, Limpsfield, £70 8s. 4d.; Chelsea Hospital for Women Convalescent Home, St. Leonards, £48 15s.; Deptford Medical Mission Convalescent Home, Bexhill, £18 8s. 4d.; Friendly Societies' Convalescent Home, Dover, £97 10s.; Mrs. Gladstone's Convalescent Home, Mitcham, £97 10s.; Hahnemann Convalescent Home, Bournemouth, £32 10s.; Hanwell Convalescent Home, £21 13s. 4d.; Hastings, Fairlight Convalescent Home, £28 3s. 4d.; Hendon, Ossulston Home, £61 15s.; Herbert Convalescent Home, Bournemouth, £32 10s.; Herne Bay Baldwin-Brown Convalescent Home, £65; Homœopathic Hospital Convalescent Home, Eastbourne, £21 13s. 4d.; Hemel Hempstead Convalescent Home, £34 13s. 4d.; Incorporated Morley and Bevan Convalescent Home, no application; Mrs. Kitto's Convalescent Home, Reigate, £54 3s. 4d.; London Hospital Convalescent Home, Tankerton, £78; Mary Wardell Convalescent Home for Scarlet Fever, £65; Police Seaside Home, Brighton, £48 15s.; St. Andrew's Convalescent Home, Clewer, £108 6s. 8d.; St. Andrew's Convalescent Home, Folkestone, £195; St. John's Home for Convalescent and Crippled Children, Brighton, £27 1s. 8d.; St. Joseph's Convalescent Home, Bournemouth, £65; St. Leonards-on-Sea Convalescent Home for Poor Children, £108 6s. 8d.; St. Mary's Convalescent Home, Shortlands, £23 16s. 8d.; St. Michael's Convalescent Home, Westgate-on-Sea, £37 18s. 4d.; Seaside Convalescent Hospital, Seaford, £184 3s. 4d.

### 26 COTTAGE HOSPITALS.

Acton Cottage Hospital, £81 5s.; Beckenham Cottage Hospital, £86 13s. 4d.; Billingsgate Medical Mission Hospital, £54 3s. 4d.; Blackheath and Charlton Cottage Hospital, £86 13s. 4d.; Bromley, Kent, Cottage Hospital, £135 8s. 4d.; Bushey Heath Cottage Hospital, £39; Canning Town Cottage Hospital, £70 8s. 4d.; Chislehurst, Sidcup, and Cray Valley Cottage Hospital, £70 8s. 4d.; Coldash Cottage Hospital, £24 18s. 4d.; East Ham Cottage Hospital, £81 5s.; Eltham Cottage Hospital, £59 11s. 8d.; Enfield Cottage Hospital, £70 8s. 4d.; Epsom and Ewell Cottage Hospital, £55 5s.; Hounslow Cottage Hospital, £43 6s. 8d.; Kingston, Victoria Hospital, £75 16s. 8d.; Livingstone Dartford Cottage Hospital, £99 13s. 4d.; Mildmay Cottage Hospital, nil; Reigate and Redhill Cottage Hospital, £119 3s. 4d.; Sidcup Cottage Hospital, £43 6s. 8d.; Sutton Cottage Hospital, nil; Tilbury (Passmore Edwards) Cottage Hospital, £54 3s. 4d.; Willesden Cottage Hospital, £119 3s. 4d.; Wimbledon Cottage Hospital, £59 11s. 8d.; Wimbledon South Cottage Hospital, £59 11s. 8d.; Wood Green Cottage Hospital, £57 8s. 4d.; Woolwich and Plumstead Cottage Hospital, £54 3s. 4d.

### 14 INSTITUTIONS.

Hospital for Invalid Gentlewomen, Harley Street, £108 6s. 8d.; St. Saviour's Hospital and Nursing Home, £108 6s. 8d.; National Sanatorium for Consumption, Bournemouth, £108 6s. 8d.; Invalid Asylum, Stoke Newington, £39; Firs Home, Bournemouth, £37 18s. 4d.; St. Catherine's Home, Ventnor, £27 1s. 8d.; Friedenheim Hospital for the Dying, £341 5s.; Free Home for Dying, Clapham, £130; St. Luke's House, Pembridge Square, W., £130; Santa Claus Home, Highgate, £67 3s. 4d.; Royal Mineral Water Hospital, Bath, £54 3s. 4d.; St. Winifred's Home, Holloway, £61 15s.; Eversfield, St. Leonards, nil; Alexandra Home, St. Leonards, nil.

### 58 DISPENSARIES

Battersea Provident Dispensary, £140 16s. 8d.; Blackfriars Provident Dispensary, £21 13s. 4d.; Blomsbury Provident Dispensary, £16 5s.; Brixton, &c. Dispensary, £54 3s. 4d.; Brompton Provident Dispensary, £18 8s. 4d.; Buxton Street Dispensary, £21 13s. 4d.; Camberwell Provident Dispensary, £86 13s. 4d.; Camden Town Provident Dispensary, £16 5s.; Chelsea, Brompton and Belgrave Dispensary, £48 15s.; Chelsea Provident Dispensary, £10 16s. 8d.; Childs Hill Provident Dispensary, £17 6s. 8d.; City Dispensary, £59 11s. 8d.; Clapham General and Provident Dispensary, £26; Deptford Medical Mission, £28 3s. 4d.; Eastern Dispensary, £41 3s. 4d.; East Dulwich Provident Dispensary, £46 11s. 8d.; Farringdon General Dispensary, £47 13s. 4d.; Finsbury Dispensary, £54 3s. 4d.; Forest Hill Provident Dispensary, £41 3s. 4d.; Greenwich Provident Dispensary, £36 16s. 8d.; Hackney Provident Dispensary, £19 10s.; Hampstead Provident Dispensary, £67 3s. 4d.; Holloway and North Islington Dispensary, £35 15s.; Islington Dispensary, £65; Islington Medical Mission, £43 6s. 8d.; Kennington and Vauxhall Provident Dispensary, £15 3s. 4d.; Kensal Town Provident Dispensary, £17 6s. 8d.; Kentish Town Medical Mission, no application; Kilburn, Maida Vale and St. John's Wood Dispensary, £40 1s. 8d.; Kilburn Provident Medical Institution, £54 3s. 4d.; London Dispensary, Spitalfields, £18 8s. 4d.; London Medical Mission, Endell Street, £131 1s. 8d.; Margaret Street, for Consumption, £27 1s. 8d.; Metropolitan Dispensary, £69 6s. 8d.; Mildmay Medical Mission Dispensary, £23 16s. 8d.; Notting Hill Provident Dispensary, £10 16s. 8d.; Paddington Provident Dispensary, £32 10s.; Public Dispensary, Drury Lane, W.C., £43 6s. 8d.; Queen Adelaide's Dispensary, £31 8s. 4d.; Royal General Dispensary, £29 5s.; Royal Pimlico Provident Dispensary, £52; Royal South London Dispensary, £49 16s. 8d.; St. George's, Hanover Square, Dispensary, £29 5s.; St. John's Wood Provident Dispensary, £53 1s. 8d.; St. Marylebone General Dispensary, £48 15s.; St. Pancras and Northern Dispensary, £34 13s. 4d.; St. Pancras Medical Mission, £22 15s.; South Lambeth, Stockwell, and North Brixton Dispensary, £43 6s. 8d.; Stamford Hill, Stoke Newington, Dispensary, £65; Tower Hamlets Dispensary, £50 18s. 4d.; Walworth Provident Dispensary, £18 8s. 4d.; Wandsworth Common Provident Dispensary, £16 5s.; Westbourne Provident Dispensary, £19 10s.; Western Dispensary, £73 13s. 4d.; Western General Dispensary, £120 5s.; Westminster General Dispensary, £47 13s. 4d.; Whitechapel Provident Dispensary, £37 18s. 4d.; Woolwich Provident Dispensary, £31 8s. 4d.

### 27 NURSING ASSOCIATIONS.

Belvedere and Abbey Wood, £20 4s.; Brixton, £30 12s.; Central St. Pancras, £22 19s.; Chelsea and Pimlico, £30 12s.; Hackney, £30 10s.; Hammersmith and Fulham, £71 4s.; Hampstead, £32 19s.; Isleworth, £15 6s.; Kensington, £68 17s.; Kilburn, £15 6s.; Kingston, £43 5s.; Metropolitan (Bloomsbury), £76 10s.; St. Olave's (Bermondsey), £45 18s.; Paddington and Marylebone, £53 11s.; Plaistow, £122 8s.; Rotherhithe, £15 6s.; Shoreditch, £96 16s.; Silvertown, £22 19s.; South London (Battersea), £78 17s.; Tottenham, £17 13s.; Southwark, £30 12s.; South Wimbledon, £35 8s.; Westminster, £30 12s.; Woolwich, Plumstead and Charlton, £55 14s.; East London, £244 4s.; North London, £84 3s.; London District, £459.

